# Delivering High-Quality, Equitable Care in India: An Ethically-Resilient Framework for Healthcare Innovation After COVID-19

**DOI:** 10.3389/fpubh.2021.640598

**Published:** 2021-02-18

**Authors:** Ahmad Ozair, Kaushal Kishor Singh

**Affiliations:** Faculty of Medicine, King George's Medical University, Lucknow, India

**Keywords:** global health (MeSH [H02.403.371]), health policy, health planning [MeSH], public health, community medicine, digital health (eHealth), digital health

## Abstract

Developing countries struggle to provide high-quality, equitable care to all. Challenges of resource allocation frequently lead to ethical concerns of healthcare inequity. To tackle this, such developing nations continually need to implement healthcare innovation, coupled with capacity building to ensure new strategies continue to be developed and executed. The COVID-19 pandemic has made significant demands of healthcare systems across the world—to provide equitable healthcare to all, to ensure public health principles are followed, to find novel solutions for previously unencountered healthcare challenges, and to rapidly develop new therapeutics and vaccines for COVID-19. Countries worldwide have struggled to accomplish these demands, especially the latter two, considering that few nations had long-standing systems in place to ensure processes for innovation were on-going before the pandemic struck. The crisis represents a critical juncture to plan for a future. This future needs to incorporate a vision for the implementation of healthcare innovation, coupled with capacity building to ensure new strategies continue to be developed and executed. In this paper, the case of the massive Indian healthcare system is utilized to describe how it could implement this vision. An inclusive, ethically-resilient framework has been broadly laid out for healthcare innovation in the future, thereby ensuring success in both the short- and the long-term.

## Introduction

“There seems to be no limit to the possibilities of scientific medicine, and while philanthropists are turning to it as to the hope of humanity, philosophers see…a science from which may come…peace over all the earth (1).” Verily, what faith in modern medicine had been expressed by Sir William Osler in 1902, is but reflected in the heroic demands made of it today—to touch more lives than ever before. Such demands warrant innovation at scale and in all aspects of healthcare. Nowhere are they more visible today, than in the worldwide call-to-arms to provide breakthrough treatments and vaccination for COVID-19 (2).

Medicine has had a long history of both scientific and social innovation. The latter, for instance, is evident in the achievements of public health, including in the current pandemic. Therefore, while ranking innovations with the greatest impact on healthcare, the Deloitte Center for Health Solutions used the following, wide-ranging definition:

“Any combination of activities or technologies, that breaks existing performance trade-offs in the attainment of an outcome, in a manner that expands the realm of the possible; defined in health care as providing ‘more for less’—more value, better outcomes, greater convenience, access, and simplicity; all for less cost, complexity, and time required by the patient and the provider, in a way that expands what is currently possible (3).”

Attainment of these presumed endpoints is what is expected of healthcare systems across the world, both in developing and developed nations, and both in the current crisis and beyond. This attainment may be either evolutionary, brought on by gradual advances, or revolutionary, which is creative, disruptive, and discontinuous. Such endpoints require one to have, firstly, a short-term strategy for translating innovative strategies into practice; and secondly, a long-term vision and philosophy for future innovation.

Analysis of a single healthcare system, in the context of its unique challenges, provides a foundation to draw lessons from. In this commentary, a broad framework of innovation for the healthcare system in India has been described, which is inclusive, ethically-resilient, and incorporates learning points from patient care experiences at the largest residential hospital in South Asia, both before and during the COVID-19 pandemic (4) (Table 1). As discussed below, rectifying health disparities, which have been exposed by the pandemic, and ensuring universal healthcare access must become India's guiding lights for health policy moving forwards.

**Table 1:** Broad action items and strategies for healthcare innovation in India after COVID-19.

**Short-term strategies**		**Long-term strategies**		
		**Pillar 1: creation of ecosystems and incentives**	**Pillar 2: manageable risk-taking**	**Pillar 3: capacity building and biomedical education**
Greater public investment	Big data and artificial intelligence	Simplifying regulations and easing creative collaborations	Running of multiple pilot projects	Enhancements in standard pedagogy
Public-private partnership	Targeted therapy or Precision medicine	Developing innovation centers, bio-incubators, and med-tech park	Decentralization of research funding	Engaging professional medical societies
Deep learning and Machine learning	“Local” medical device manufacturing	Ensuring adequate compensation for biomedical researchers	Allowing personal and organizational failures without immediate shutdown	Physician Scientist (MBBS-PhD) and masters in biomedical innovation programs
Telehealth and remote patient monitoring	Utilizing available devices (smartphones) for healthcare delivery	Creating cultures of innovation—less hierarchy, more communication	Promoting social innovation and entrepreneurship	Creating medical schools having purposeful aim of biomedical innovation

## The Challenge in India

Even before COVID-19 struck, the majority of the Indian population was yet to have access to healthcare in its entirety, let alone an excellent one. The public sector was already contributing merely 30% to the cost here, in contrast to 84% in, for instance, the United Kingdom (5). Out-of-pocket expenditure made up over 60% of total healthcare expenses, in a nation having 273 million living below the international poverty line of US$1.90 per person per day (6). Coupled with the average public spending on healthcare per capita, a meager INR 3/- per day, the scale of the economic challenge becomes clear (5). Unfortunately, the above challenge has only been accentuated by the pandemic, with estimates allowing for a possibility of doubling of poverty (7, 8). Issues of unavailability of critical medical equipment and unaffordability of the private sector have also been brought to the fore—issues that can be tackled by innovation and reform as well (9, 10).

A nation that ranks amongst the lowest worldwide for the percentage of its gross domestic product (GDP) spent on healthcare, therefore, requires “frugal innovation,” driven by prioritized analysis of population-level healthcare needs. The intent to implement innovation must be with a razor-sharp focus on ensuring that benefits flow to the bottom of the pyramid. In planning for India's future, it must be remembered that even emergency care remains an elusive entity for most, let alone annual health check-ups (11).

Before the COVID-19 pandemic, the biggest killers in India, as per the World Health Organization, in decreasing order of frequency, had been ischemic heart disease, chronic obstructive pulmonary disease, stroke, diarrheal disease, lower respiratory infection, preterm birth complications, and tuberculosis (12). What binds together the top three in this list, together causing a third of all deaths, are the chronic nature of their risk factors, i.e., diabetes, hypertension, dyslipidemia, smoking, visceral obesity, etc. All of these are modifiable or preventable, and thus well-positioned to be the arena of healthcare innovators.

## Short-Term Strategies for Innovation: The Current Big Ideas

Short-term strategies for innovation in India must employ both top-down and bottom-up approaches to succeed, merely one does not succeed alone. The top-down strategy needs a larger number of more enhanced streamlined public-private partnerships, along with a greater public investment in biomedical research. While the latter has been seen in the recent funding provided for developing therapeutics and vaccines against COVID-19, this support needs to be sustained, directed, and unconditional, similar to how the Indian space and aeronautics agencies were provided in their early days. Thus for healthcare, this funding must not merely be contingent on producing a regular stream of publications. The bottoms-up strategy, for instance, needs our finest medical graduates stepping forward for biomedical innovation—the vast majority of them currently pursue clinical practice alone or migrate to greener pastures, where significant incentives for biomedical research exist.

Notably, the twenty-first century is blessed with vast stores of immense, deep, accessible, and ever-growing knowledge, i.e., big data, which can be utilized with great impact in medicine (13). With the explosion of monitoring devices and electronic health records globally, as researchers get access to considerably more patient data than ever before, many conclusions are yet to be drawn by predictive analytics. If crunched with pattern recognition, big data can provide specific, especially regional, epidemiological information, and differential therapeutic responses (14).

Tectonic plates underneath classical healthcare have long been shifting; COVID-19 has greatly accentuated their movement. Particularly, the technologies of telehealth, remote patient monitoring, and consulting by mobile technology (mConsulting), had been leisurely chugging on prior—they have now been thrust into full view because of necessity (15, 16). In our institution, an Electronic COVID Care Support (ECCS) system, where a multidisciplinary team of pulmonologists, intensivists, and internists were available round the clock to advise and guide intensive care units across the province in managing complex COVID-19 patients, especially those with multi-organ dysfunction. This was of crucial value in our province, where fellowship-trained intensive care physicians are but a handful and that too nearly all in the provincial capital. Similarly, the city administration constructed a call center for medical queries from the general public which junior doctors from our institution staffed, resulting in significant benefit to the general public with regards to managing mild COVID-19 cases at home. Considering how useful such technologies have proved, even in India where the smartphone penetration rate has been rapidly growing, usage of these technologies will likely continue after the pandemic as well (16).

Using machine learning (ML), advanced image-processing capabilities enhanced by artificial intelligence (AI), convolutional neural networks (CNN), deep learning (DL), natural language processing (NLP), many clinically-proven technologies have been described, some approved by regulatory bodies, and a number are rapidly forthcoming (17). Coupling image processing with ML/DL has been especially useful in visual-heavy fields like dermatology, pathology, ophthalmology, and radiology (18). For instance, Arterys Cardio DL, the cloud analytics software recently approved by the U.S. Food and Drug Administration (FDA), helps interpret cardiac MRI, reduces the workload of radiologists, and thus significantly decreases the need for specialists (19).

How will these technologies be implemented in India? Consider a use-case scenario of the “CheXNeXt” algorithm (20). This CNN-based technology utilizes chest x-rays, the most common imaging tool across the world. Built by researchers at Stanford University, it has been proven to diagnose pneumonia in suspected cases better than radiologists of the same institution, historically long-considered one of the top-ten best radiology training centers in the US (21). India, on the other hand, with its immense burden of infectious diseases, sorely lacks radiologists, especially at primary and secondary healthcare centers. However, x-ray diagnostic facilities are often available at community health centers. Therefore, images taken here may be uploaded online for analysis by the algorithm resulting in guidance for the rural physician. This will allow rapid confirmation of the diagnosis in non-complex cases, the majority, by a basic medical doctor leading to an early start of empiric treatment and better patient outcomes. Such innovations, by radically reducing the number of specialists required, hold immense hope for healthcare delivery in the most remote villages. Technology here complements clinical competency, with the algorithm diagnosing the majority of cases and flagging the challenging ones for review and resolution by the expert. Thus, implementing CheXNeXt in a nation where over two-thirds live in villages can have a significant impact (22). Unfortunately, support for this technology was not available in the current pandemic in the majority of India, where doctors in rural areas could have benefitted by getting X-rays of suspected COVID-19 pneumonia cases evaluated by a radiologist or a counterpart.

Furthermore, as part of healthcare innovation, AI can not only diagnose disease under supervision as above; it has been proven to work without the need for a supervising doctor, in selected cases. This gains special relevance in a nation having one of the poorest physician (allopathic) to patient ratios worldwide. Consider IBM's Watson for Oncology, whose independent therapeutic decision-making for breast cancer cases had a 93% concordance with a multi-disciplinary tumor-board (23). Similarly, “IDx Dr,” the first FDA-approved AI-based diagnostic system for screening of diabetic retinopathy, also does not require an ophthalmologist for its operation (24). Thus, it is of value to secondary healthcare centers in inaccessible areas, in India, permitting them to screen a large number of diabetics (25). This is crucial for a country having over 70 million diabetics (26), and a current epidemic of retinopathy (27). Another work, published in 2020, promises similar significance by detecting fundal papilledema with over 96% sensitivity (28).

An additional bulwark of an approach would be the leveraging commonly available devices for healthcare delivery, whose built-in technologies would also be utilized for point-of-care (POC) diagnostics. For instance, verified smartphone apps, using AI, can act as personalized, patient advisors: answering their unmet needs, helping adhere to prescribed medications, and encouraging them to implement lifestyle changes (29). This technology has already been utilized by retail industries. Here, this frees up the specialist, especially in overloaded public hospitals, to manage cases of highest acuity. In the current pandemic, while an app was launched by the federal government for responding to queries of the users, at the backend it required significant manpower to respond to the queries. Innovations using currently available tools, as used successfully prior by suicide prevention hotlines using the ubiquity of landlines, will prove critical in India, having over 300 million smartphone users (30).

Similarly, “AliveCor Kardiaband,” the recently FDA-approved sensor (31), delivers a medical-grade six-lead ECG, in conjunction with a smartwatch. The REHEARSE-AF RCT proved its clinical utility by showing that it was better able to detect incident atrial fibrillation, compared to routine care (32). As the electronics industry evolves, appliance costs fall. We believe that as prices of smartwatches compatible with this technology will likely come down, it will lead to its greater adoption by large parts of urban India, with face-to-face consultation expenditures saved and patient outcomes improved.

## The Case for More for Less

The reader may return now to one of the sections in the definition of healthcare innovation, used previously, of “more for less.” Innovation in India, therefore, must also include efforts to reduce the costs of existing technologies that are currently either inaccessible or pose a considerable challenge to the funding of public hospitals, such as robotic surgery, biosensors, 3D-printed prosthetics, etc. Hospitals here lack reliable alternatives from local manufacturers for equipment such as even endoscopes, let alone surgical robots, and have to purchase these at a premium from foreign manufacturers. This dearth of high-quality, medical-grade manufacturing capacity has been felt acutely in the ventilator shortage during the COVID-19 pandemic as well, with local manufacturers scrambling to develop capacity (33). Had Indian medical corporations been manufacturing ventilators with significantly high enough output and uptake in major hospitals, a period of difficulty may have been averted, where a shortage of ventilators was felt.

What is required is an Indian medical equipment industry, functioning at the level of global standards, such as the economical Jaipur foot that can give the original “SACH foot” a run for the money (34, 35). Such an industry will also help make accessible new POC testing for cancer, stroke, etc., thus greatly assisting in their testing in rural, remote, and disprivileged populations in India, leading to their detection and/or early management. One can envision this similar to the worldwide revolution brought about by the widespread access to POC pregnancy tests (36). Lessons must be learnt from how empowering is the availability of medical equipment in rural areas, evident in the success of “foldscope” in diagnosing parasitic diseases in resource-limited settings (37). Verily, this was a challenge in the current pandemic considering that the reverse transcriptase-polymerase chain reaction (RT-PCR) based testing for COVID-19 could only be carried out in certain laboratories, which were geographically limited in the initial parts of the pandemic. Our own institution performed the maximum number of tests carried out at any single center in India.

Moving away from a one-size-fits-all approach will be another innovation strategy. An NHS report suggests that upto 70% of patients do not benefit from the conventional treatment pathway using mass-manufactured medications (38). Notably, the cost of gene-based studies has been rapidly falling. Low cost, widely-available next-generation sequencing (NGS) and genotyping will enable the prescription of personalized drugs leading to less therapeutic failures and more consistent responses (14, 39). Early recognition of and intervention in high-risk groups also prevents significant costs later in life. This cost-effectiveness has been, for instance, well-demonstrated in familial hypercholesterolemia cases treated with evolocumab (40). As noted in a cost-effectiveness work that looked at universal genetic screening for BRCA1/2 mutations, bringing down the test cost to below 250$ resulted in the ratio of cost per quality-adjusted life-year (QALY) reaching 53,000$/QALY, well-under the commonly cited US-based threshold of 100,000$/QALY (41). A similar hope is enshrined in the 3D-printing of prosthetics tailored to the patient (42). In the current pandemic, several promising drug candidates were looked at, such as hydroxychloroquine, remdesivir, etc. but then largely abandoned due to the majority of studies demonstrating a lack of efficacy. It is useful to consider the idea of whether genotype-based analyses could have helped select patients who found have benefitted from specific agents.

Optimism is also well-placed in economical biosensors. These would ideally allow for continuous, painless, and non-invasive monitoring of vital parameters, and help guide both prevention and treatment. Unfortunately, very few devices like the fingertip pulse oximeter exist, which did have the above attributes and was a valuable tool in the current pandemic both in the home-based and the in-hospital management of COVID-19 cases. These devices are especially useful in patients where long-term monitoring is required. Unfortunately, in India, a large number of such patients are unable to remain in regular follow-up at the often distant tertiary care center due to the issues of cost, time, and lost income therein. Here, such sensors can provide data for remote follow-up and allow for greater time between physical visits. For instance, the “Lumee” biosensor allows for long-term monitoring of tissue oxygen levels, helping in the follow-up of peripheral vascular disease, which tends to also be neglected in rural areas (43).

## Is India Equipped to Implement these Strategies?

It is important to note the two major bottlenecks for these short-term innovation strategies. These are, first, possession of strong computer science and medical technology industries, and second, deep ability to carry out large clinical trials to prove efficacy. While India's historical competency in the technology arena creates a bedrock of medical innovation to be done in-house, its massive population allows for large-scale careful studies to be conducted at a much lower cost for the latter, without sacrificing any quality paradigms. This is exemplified by the largest clinical trial ever in the world, conducted in India in over two million children (44, 45).

Other limitations to implementing these strategies include the political will to devote support and manpower to enhancing healthcare and increasing the percentage of GDP spent on this sector. Discussion of how to tackle this and how to improve financing into interventions to improve social determinants of health are beyond the scope of this article.

Thus, India is uniquely poised to deliver rapid breakthroughs in its healthcare delivery, provided its research institutions and innovators are specifically tasked with and incentivized to solve this problem. Developing such “disruptive innovation” is vital, as originally described by Bower and Christensen, since it represents a stratagem different from incremental enhancements in good healthcare for those who already have it (46). As a part of a multi-faceted approach to innovation, it gives accessible healthcare to those who entirely lack it.

## The Long-Term Game-Plan

In grappling with strategies to translate strategies for innovation into practice, it is imperative to have a parallel approach to developing a system that fosters their rise and provides them with a structure to lean on to. *Three* pillars will need to be erected, upon which hopefully the edifice of innovation will transpire (Table 1).

The first pillar must be the creation of ecosystems and incentives that promote creative collaboration. Disruptive ideas of the future will emerge at the interface of different fields, as has occurred before, for instance, in the development of the CT scanner (47). This will require cross-pollination of varied concepts and processes of thought, possible only when collaboration between institutions, hospitals and industry happens in interdisciplinary areas such as data capture and analytics, patient engagement, and natural sciences, exemplified by the development of CRISPR for gene editing (48). During the pandemic in India, an example of this was shown in the development and usage of the paper-based rapid diagnostic testing for COVID-19 utilizing CRISPR, named FELUDA (49). To note, such cross-pollination can be facilitated by the creation of innovation centers, bio-incubators, and med-tech parks, some of which have recently been set-up (50).

What else can be done to ensure these creative collaborations? Simplifying and streamlining slow, bureaucratic, regulatory, and administrative processes, all of which hinder collaborative work is critical. To illustrate, consider the challenges encountered in doing multicentric studies in India—for instance, each participating center must separately apply for and complete the entire institutional review board (IRB/IEC) approval process, as per current regulations. Thus, no center can utilize the fact that a study has been already approved by another government-accredited IRB/IEC, so as to have a faster review at their own IRB/IEC. This was a major difficulty faced by our institution and others when conducting multicentric clinical trials in a time-sensitive manner for evaluating interventions for COVID-10. Hindrances such as these slowed down the pace of biomedical research in a period of urgency and contributed to the delay in getting medications approved and to the bedside. Besides this, a rectification of the strict, pervasive, and all-sustaining culture of hierarchy in medicine in India would also help. It has historically stifled collaboration, prevented voices from speaking up, and overemphasized the role of chairpersons and directors at the cost of the team members. Furthermore, cognizance must be taken of the fact that the finest achievements of medicine in the current century have been due to breakthroughs in biology and biotechnology. Scientists of these fields must be better incentivized and recognized for doing biomedical research in India. Institutions here must also further subject the wisdom of their traditional texts to rigorous research, as exemplified by the Nobel-prize-winning discovery of Artemisinin in China (51).

The second pillar must be the espousal of a philosophy that allows for a host of pilot projects to run. Our anecdotal experience suggests that senior, established investigators are heavily rewarded with grants in India, while early career researchers fail to get anything significant. A risk-averse behavior, as has been historically the Indian perspective, must be replaced with conduction of small-scale pilot projects with “manageable risks,” allowing a large number of institutions to have funds to experiment and to demonstrate “proof-of-concept.” Greater decentralization of research funding would help this as well. Herein, thought must also be given to social innovation and entrepreneurship in medicine, as demonstrated by the success of “Swachh Bharat” with regards to sanitation and hygiene (52). Similarly, considering the prevalence of domestic abuse, malnutrition, and nutritional anemia in Indian women, strategies that empower financially women also indirectly contribute to their healthcare, like the work done by Sakha Consulting Wings (53). These are often harder to implement but produce far more long-lasting impact.

The third pillar must be capacity building and reform in medical education. Firstly, pedagogic teaching of subjects in Indian medical schools today lacks both focus and relevance, since much of it is “teaching to the test.” This test is typically either the medical school examination or post-graduate medical entrance examination, both of which strongly focus on rote memorization. This adversely contributed to the current pandemic since final year medical students here could not be well-utilized to care for COVID-19 patients since students in India do not manage patients under supervision till their internship year. It is also well-documented that the content of education imparted molds itself to the testing thereafter, a phenomenon referred to as the “washback” or “backwash” effect (54, 55). Improvements in testing methodology will, therefore, likely result in the enhancement of medical education as a backwash effect. Thus, to realize the intent of imparting scientific curiosity and a spirit of innovation, examinations must become tailored to test relevant issues, coupled with a deliberate attempt at gradual modernization of the entire curriculum (55). Professional medical societies in India must become engaged in this modernization, in addition to having them connecting with and academically mentoring their future trainees, similar to their counterparts in the US and UK.

Furthermore, it would be helpful to start joint physician-scientist (MBBS-PhD) programs, similar to the medical scientist training programs (MSTP), also known as MD/PhD programs, in the US. Otherwise, an MBBS (Honors) or an MBBS with an intercalated research-based BSc (as is common in the UK) program may be created at apex medical schools. Certain premier medical schools of India may also be designated as those having a novel curriculum merging classical medical training with biomedical innovation. None of these four options currently exists in India. Similarly, an increase is also warranted in the number of institutions offering masters programs in medical innovation, currently, there being few such in the country, for instance, the quite successful Masters in Medical Science and Technology (MMST) program at Indian Institute of Technology Kharagpur, which solely takes in medical school graduates (56).

All of these programs must purposefully aim to create future-ready graduates in fields of innovation, interdisciplinary research, technology advancements, data analytics, statistical capabilities, etc. These programs would mandate additional courses on computer science, molecular biology, rapid prototyping, and/or medical material science, courses that are not part of the current medical school curriculum in India. Finally, these programs must also be in-line with the aspirations of our medical graduates and the health needs of the country, deliberately focusing on “frugal innovation.”

## Conclusion

Healthcare innovation in India will be brought about by a multipronged approach: early-attainable, specific objectives of implementing efficacious new strategies for the priority healthcare needs of the population, i.e., frugal innovation; and a long-term vision for fostering the culture that promotes such advancements; or else worthy intentions will keep getting lost in translation.

## Author Contributions

AO conceptualized, drafted, and edited the manuscript. KS edited the manuscript. Both authors contributed to the article and approved the submitted version.

## Conflict of Interest

The authors declare that the research was conducted in the absence of any commercial or financial relationships that could be construed as a potential conflict of interest.
